# A Comparison of Physical Characteristics in Different Brands and Staining Techniques in a Brand of Lissamine Green Strips

**DOI:** 10.3390/jcm14062022

**Published:** 2025-03-17

**Authors:** Neema Ghorbani-Mojarrad, James S. Wolffsohn, Jennifer P. Craig, Debarun Dutta, Byki Huntjens, Raheel Hussain, Zarghona Khan, Shoaib Raja, Mohammed Ibrahim, Thomas Godfrey, Alison Alderson, Katharine Evans, Mahesh Joshi, Carole Maldonado-Codina, Manbir Nagra, Elidh Martin, Laura Sweeney, Louise Terry, Dean Dunning, Marta Vianya-Estopa

**Affiliations:** 1 School of Optometry and Vision Science, University of Bradford, Bradford BD7 1DP, UK; n.ghorbanimojarrad@bradford.ac.uk (N.G.-M.);; 2 Wolfson Centre for Applied Health Research, Bradford Royal Infirmary, Bradford BD9 6RJ, UK; 3 School of Optometry, Aston University, Birmingham B4 7ET, UK; 4 Department of Ophthalmology, Aotearoa New Zealand National Eye Centre, The University of Auckland, Auckland 1010, New Zealand; 5 Department of Optometry and Visual Sciences, City St George’s, University of London, London WC1E 7HU, UK; 6 School of Optometry and Vision Sciences, Cardiff University, Cardiff CF24 4HQ, UK; 7 School of Health Professions, University of Plymouth, Plymouth PL4 8AA, UK; 8 Faculty of Biology, Medicine and Health, The University of Manchester, Manchester M13 9PR, UK; 9 Vision and Eye Research Institute, Anglia Ruskin University, Cambridge CB1 1PT, UK; 10 Department of Vision Sciences, Glasgow Caledonian University, Glasgow G4 0BA, UK; 11 School of Ophthalmic Dispensing, Bradford College, Bradford BD7 1AY, UK; 12 Vision and Hearing Sciences Research Centre, Anglia Ruskin University, Cambridge CB1 1PT, UK

**Keywords:** lissamine green, bulbar conjunctiva, staining, ophthalmic dye, physical characteristics, technique

## Abstract

**Backgrounds/Objectives:** The aim of this study was to compare differences in the physical characteristics of lissamine green (LG) strips and the outcomes of using different staining techniques. **Methods:** Two separate complementary investigations were conducted. Physical study: Differences between four LG strips were evaluated in terms of material, dye concentration, and dye absorption. In vivo study: Bulbar conjunctival staining was compared for four application methods of I-DEW LG strips presented in a randomized order for twenty-two participants: (1) single application 5 s after wetting (also repeated using GreenGlo for comparison), (2) single application using two strips held together, 5 s after wetting, (3) two applications using a single LG strip 5 s after wetting, 1 minute apart, (4) the same as method 3, with a single fluorescein strip in between LG applications. White light imaging was performed immediately following application and after 30, 60, 90, and 300 s. Three masked practitioners independently evaluated the randomized staining images for spot count and staining intensity. **Results:** Physical study: Strip paper fibres demonstrated visible similarities, with no difference in saline absorption (*p* > 0.05). LG concentration increased as saline retention duration increased (F = 964.1, *p* < 0.001), and GreenGlo tips were significantly darker (F = 2775.2, *p* < 0.001). In vivo study: I-DEW application resulted in less conjunctival staining than GreenGlo (*p* < 0.001). Amongst I-DEW application techniques, staining levels were similar (*p* > 0.05); however, staining intensity was significantly higher following two applications of I-DEW, 1 min apart, compared to a single application (*p* = 0.042). Both spot count and staining intensity decreased with time (*p* < 0.001). **Conclusions:** Two applications of I-DEW using a single strip, 1 min apart, after wetting with a single drop of saline provided maximal staining. There was also a significant difference in staining intensity observed between LG products.

## 1. Introduction

Lissamine green is an effective dye for identifying changes to the bulbar conjunctiva and lid margin that occur with ocular surface disease. It is therefore used in dry eye assessment in eye care to evaluate conjunctival staining, both for diagnosis and monitoring [1]. Lissamine green has also been used to look at the lid wiper region for signs of lid wiper epitheliopathy and is the preferred method for assessing bulbar conjunctival staining [2]; it is well tolerated by patients and has a good safety profile at several different concentrations [3].

Lissamine green is most commonly available in the form of impregnated paper strips which are hydrated with saline in order to apply the dye to the ocular surface. Although lissamine green can sometimes be obtained in liquid drop form (or diluted in clinical practice to achieve this), this is not easily available in many parts of the world, including the UK, where impregnated strips are primarily used. These strips can vary, with a clinical study reporting that several brands of lissamine green strips (all marketed as 1.5 mg impregnated strips) produce different amounts of visible staining when examining the lid wiper region [4].

TFOS DEWS II recommended “a lissamine green strip is wet with saline, with the whole drop retained on the strip for at least 5 s to elute the dye” [1] as studies had found the optimal volume to be 10 µL [5] of 1% lissamine green solution [6]. A more recent study involving a single brand of lissamine green (GreenGlo) examined the optimum methodology for the assessment of bulbar conjunctival lissamine green staining, including the impact of the duration of saline drop retention on a lissamine green strip on dye concentration. The study showed that retaining the drop on the strip for 5 or 10 s outperformed immediate application of lissamine green with regard to bulbar conjunctival staining intensity [7]. Application method and drop retention therefore have a significant impact on the amount of staining observed. However, it is currently unknown whether these findings apply during the application of other branded strips, given the inter-product differences reported [4]. In a study where the dye was retained on the strip for 5 s and the excess shaken off before application, staining was found to differ between two regulated lissamine green strips, despite containing the same stated concentration of lissamine green (1.5 mg) [8]. This implies that different products create different levels of staining, meaning that staining results from one product may not necessarily be consistent with one another; it also implies that investigation of different products using different application techniques is beneficial. In addition, the study exclusively investigated staining on the lid wiper region [4], meaning that the differences in lissamine green products for producing staining on the bulbar conjunctiva have not been investigated.

In light of the reported inconsistencies, this work aimed to investigate physical and clinical characteristics for different lissamine green strips using two separate but complementary investigations. The physical characteristics of the lissamine green strips were assessed by examining the strip material of four different lissamine green strip products: BioLissamine (Biotech Healthcare Group, Ahmedabad, India), GreenGlo (HUB Pharmaceuticals Ltd., Scottsdale, AZ, USA), I-DEW (Entod Research Cell, London, UK Ltd., London, UK), and OPTITECH Lissamine Green (Optitech, Prayagraj, Uttar Pradesh, India). On-eye performance was assessed with an in vivo study according to spot count and the intensity of bulbar conjunctival staining seen when applied with a range of application methods and observation times post dye instillation using two of the aforementioned products, namely I-DEW and GreenGlo lissamine green strips (which are licenced for use in the UK). Together, these investigations will help determine whether differences exist across brands and identify the optimal approach for assessing bulbar conjunctival staining using different brands of lissamine green strips.

## 2. Materials and Methods

This work investigated the performance of different lissamine green-impregnated strips through two different study designs.

### 2.1. Physical Characterization Study

Four commercially available lissamine green strips were evaluated:

- BioLissamine, Biotech Healthcare Group, Ahmedabad, India;
- GreenGlo Lissamine green Ophthalmic Strip, HUB Pharmaceuticals Ltd., Rancho Cucamonga, CA, USA;
- I-DEW GREEN Lissamine green Strips, Entod Research Cell UK Ltd., London, UK;
- OPTITECH Ophthalmic Diagnostic Strip Lissamine green Strips, Uttar Pradesh, India.

The lissamine green samples were prepared using a glass slide that was placed on a digital scale and the scale tared (using a fresh slide each time). A Gilson pipette (20–200 µL capacity) was used to apply 40 µL of Sensitive Eyes Plus Saline (Bausch + Lomb, Rochester, NY, USA) to the centre of the green section of the lissamine strip and after the required drop retention time (0 s, 5 s or 10 s), the strip was touched onto the glass slide. For the ‘wash’ condition (to determine how much lissamine could be clinically extracted if the strip had been ‘squirted’ with saline rather than a drop applied), the surface of the strip tip was ‘rinsed’ for 2 s in saline over a kidney dish and immediately touched onto a slide. The difference in slide weight with the additional drop was calculated and the slide was placed between a broad visible spectrum light emitting diode panel (background light then subtracted) and a spectrometer (Ocean Optics, Dunedin, FL, USA) to determine the transmission profile of the resulting drop. Each brand and condition was assessed 3 times in randomized sequence and averaged. The tip and edge were also imaged (dry) with a 60–120 times LED digital microscope (Carson MM-300 MicroBrite plus, Carson Optical, Ronkonkoma, NY, USA) to observe and quantify the colour, dimensions, and paper fibre density (using ImageJ, version 1.54 NIH, Bethesda, MD, USA).

### 2.2. In Vivo Study

A researcher-masked in vivo study was performed at two sites (Aston University and the University of Bradford) within the UK. This study was granted approval from the Research Ethics Committees at the University of Bradford and at Aston University. All participants provided full informed consent to participate, with this study conforming to the tenets of the Declaration of Helsinki.

Participants were recruited locally at both sites through emails, word of mouth, and posted advertisements. Inclusion criteria required participants to be at least 18 years old and able to provide full informed written consent. The inclusion criteria included dry eye symptomatology (measured using a 5-item Dry Eye Questionnaire [DEQ-5] score ≥ 6) [9] and visible conjunctival staining after the application of lissamine green (with a full drop of saline applied after it had been retained on the strip tip for 5 s). Wearers of soft contact lenses were eligible to participate, but were asked not to wear their lenses for 24 h prior to each study visit. The exclusion criteria included wear of rigid corneal or scleral lenses, those with known adverse reactions or allergies to topical ophthalmic drugs or dyes used in this study, or anyone who was, or could potentially be, pregnant or breastfeeding or had any anterior eye surface disease (that was not dry eye-related).

Eligible participants attended five visits, each at least 24 h apart, with a different dye application technique randomly assigned at each visit. All visits were scheduled for a similar time of day (within ±1 h of each other) for each participant to mitigate any possible diurnal effect on ocular surface staining. Two brands of lissamine green were approved for clinical use in the UK at the time of the study, I-DEW and GreenGlo. To allow for comparison with previous findings [7], GreenGlo was compared, as a control product, to I-DEW under a single experimental condition consisting of a single application of dye 5 s after wetting the strip with a single drop of Sensitive Eyes Plus saline (Bausch + Lomb, Rochester, NY, USA). Three other application methods were investigated with the I-DEW product only, expanding on the application options explored previously [7]:

1. A single application of I-DEW lissamine green, using two strips placed together, 5 s after wetting the strips with a single drop of saline;
2. Two applications of I-DEW lissamine green, one minute apart using the same strip, 5 s after wetting with a single drop of saline each time, with an application of fluorescein (BioFluoro, 1.0 mg; Biotech Vision Care, Secunderabad, India) moistened with a saline drop with the excess shaken off, in between applications;
3. Two applications of I-DEW lissamine green, one minute apart using the same strip, 5 s after wetting with a single drop of saline each time.

Application and imaging were conducted on the right eye only. Images of the temporal bulbar conjunctiva were captured with 10 timesmagnification using a digital slit lamp biomicroscope at the following time intervals:

1. Immediately after application;
2. Thirty seconds after application;
3. Sixty seconds after application;
4. Ninety seconds after application;
5. Three hundred seconds after application.

Images were randomized by an independent member of the research team, who sent the images to three eye care professionals (qualified for between 20 and 32 years) who graded the staining observed in each of the images. Graders were not involved in data acquisition or analysis to avoid potential bias and worked independently from one another.

Quantification of conjunctival staining by graders was performed using two metrics: numerical counting of the visible spots of staining present and grading the intensity of staining. The creation and use of a visual grading scale to quantify lissamine green conjunctival staining intensity has previously been reported by the same research group [7] and was also used for this study. The grading scales were provided in printed copy format to each of the graders to allow them to conduct the task of grading all the images for lissamine green intensity whilst keeping the scale in front of them for reference. Once graded, the images and their grades were sent back to the researcher who reordered them into dye/product application and time order for each participant to allow for statistical analysis.

### 2.3. Data Analysis

Data analysis was performed in SPSS Statistics (version 26; IBM, Armonk, NY, USA). As the data did not differ significantly from a normal distribution (Kolmogorov–Smirnov test, *p* > 0.05), a two-way repeated-measures analysis of variance (ANOVA) was used to determine whether there was a significant difference in either the number of spots counted or the lissamine green staining intensity over time. Where statistical differences were found, post hoc paired *t*-tests were applied. Correlations between the number of punctate spots and their graded intensity was performed using Spearman’s Rank. A *p* value of <0.05 was set as the threshold for statistical significance.

## 3. Results

### 3.1. Physical Study

**Figure 1:**
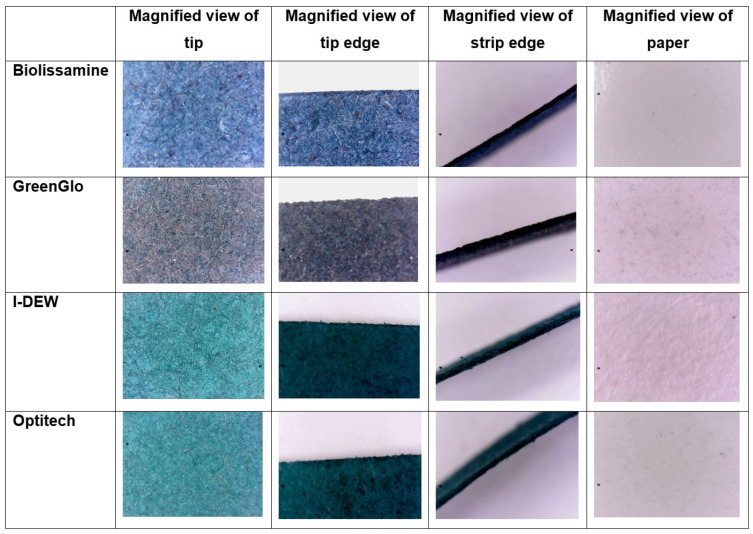
Images of magnified paper strip tips and edges (at 120 times magnification) and paper of the body of the strip and its edge (at 60 times magnification) for each of the different lissamine green brands under investigation in the physical study.

The relative density of tip fibres (BioLissamine 82.5 ± 2.6%; GreenGlo 82.0 ± 0.2%; I-DEW 77.5 ± 0.2%; OPTITECH 62.0 ± 0.3%) and the paper handle (BioLissamine 0.7 ± 0.5%; GreenGlo 3.5 ± 0.1%; I-DEW 5.6 ± 0.3%; OPTITECH 0.5 ± 0.1%) of 1.5 mg of impregnated lissamine green strips werelargely similar in appearance, whereas the hue (green compared to overall density) at the tip that was impregnated with dye differed (BioLissamine 31.8 ± 0.3%; GreenGlo 32.1 ± 0.2%; I-DEW 38.7 ± 0.2%; OPTITECH 38.7 ± 0.2%; Figure 1).

**Figure 2:**
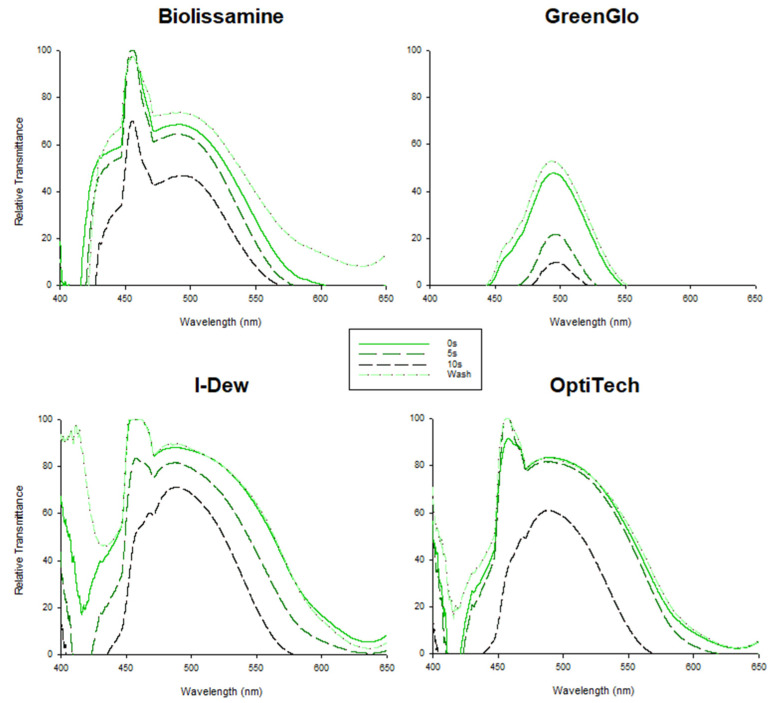
Spectral profile of lissamine green strip tip with different saline drop exposure times for the different brands.

**Figure 3:**
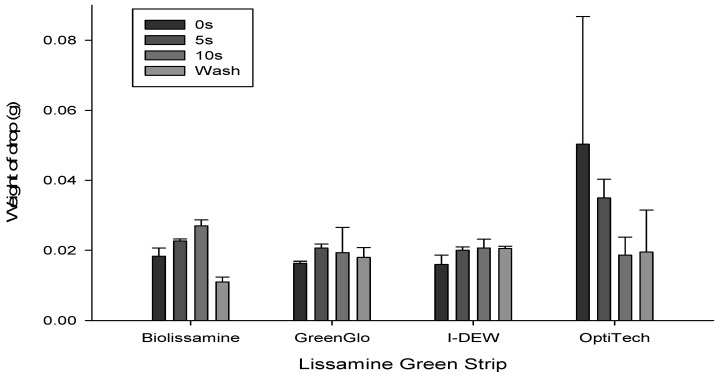
Drop of lissamine green weight after saline had been applied to the strip tip for each exposure time for the different brands.

For all the strips under investigation, washing the tip offered a slightly lower lissamine green concentration relative to immediate application (F = 3432.152, *p* < 0.001), whereas the concentration that would be applied to the eye was found to increase as the duration of retention of the saline on the strip tip increased from 0 to 10 s (F = 964.070, *p* < 0.001; Figure 2). The GreenGlo solution was significantly darker (a lower transmission across wavelengths, implying higher concentration) than the other three brands (F = 2775.200, *p* < 0.001; Figure 2). Drop weight (F = 0.665, *p* = 0.627) was not significantly affected by the duration of saline drop exposure to the strip tip; in addition, the weight of the drop from each brand was similar (F = 1.289, *p* = 0.460; Figure 3).

### 3.2. In Vivo Study

Twenty participants who met the inclusion criteria were recruited, completed all study visits, and were included in the analyses. The average age of participants was 22.9 ± 3.7 years old; 70% were female, with an average DEQ-5 score of 13.3 ± 5.5.

**Figure 4:**
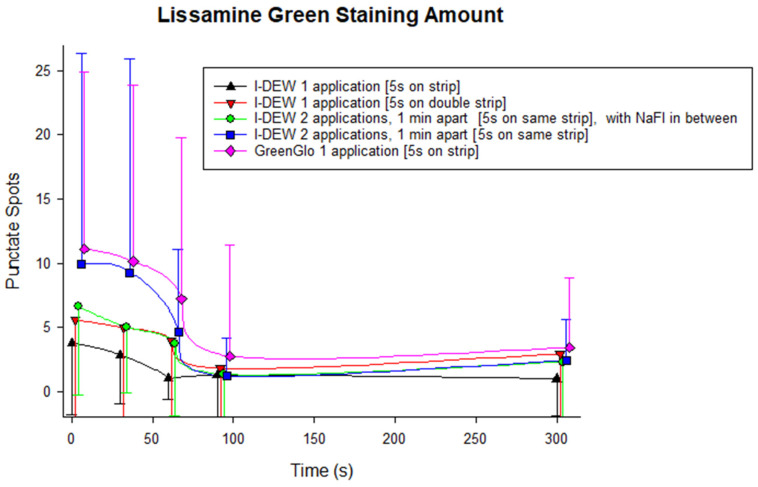
Average punctate spot count over time for the different lissamine green application methods (mean ± SD). Horizontal jitter was applied for better visualization. LG: lissamine green; NaFl: sodium fluorescein.

**Figure 5:**
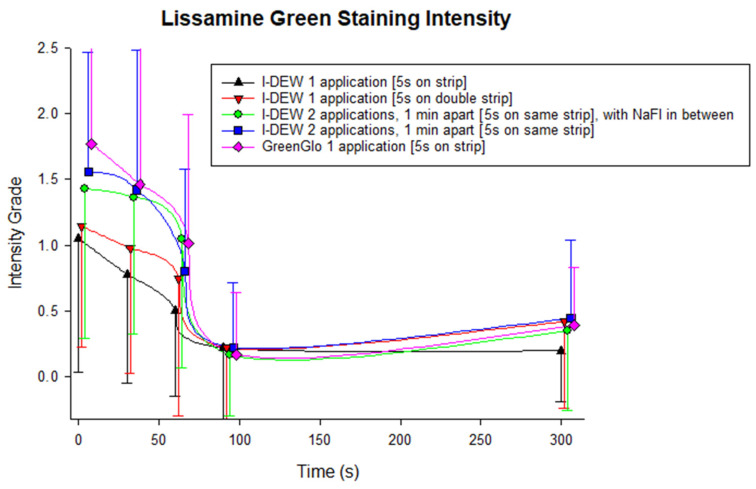
Average lissamine green staining intensity grade for the study application methods over 300 s (mean ± SD). Horizontal jitter was applied to the data points to aid visualization. LG: lissamine green; NaFl: sodium fluorescein.

GreenGlo lissamine green application resulted in the greatest amount of conjunctival staining according to both quantitative evaluations compared to all application techniques tested with the I-DEW lissamine green (Figure 4 and Figure 5), although in some instances, this was only marginal. I-DEW lissamine green dye application resulted in significantly less conjunctival staining than GreenGlo for the identical application method (i.e., single application after a drop of saline had been retained on the strip for 5 s), both in terms of the number of punctate spots (3.8 ± 5.5 vs. 11.1 ± 13.8; *p* < 0.001; Figure 4) and staining intensity (grade 1.0 ± 1.0 vs. 1.8 ± 1.3; *p* < 0.001; Figure 5).

Amongst the four I-DEW application techniques, there was no overall significant difference in punctate staining count (F = 1.979, *p* = 0.127) or staining intensity (F = 2.125, *p* = 0.106), and the staining profiles over time were similar (F = 1.034, *p* = 0.418 for staining spot count; F = 1.634, *p* = 0.083 for staining intensity). However, peak staining intensity (*p* = 0.042) and the intensity of the punctate spots at 300 s (*p* < 0.001) was significantly higher with the method that involved two applications of I-DEW 1 min apart using the same strip with the saline drop retained each time for 5 s compared to a single application.

For all application methods, both the spot count (F = 10.862, *p* < 0.001) and staining intensity (F = 25.616, *p* < 0.001) decreased with time, with the greatest amount of conjunctival staining seen immediately after application. There was no evidence of an interaction effect between viewing time and method of application for measures of staining spot count, or staining intensity (*p* > 0.05).

The correlation between spot count and staining intensity measures within all the lissamine green conjunctival staining images was 0.855 (*p* < 0.001).

## 4. Discussion

This study investigated physical differences between commercially available lissamine green strips, as well as differences in bulbar conjunctival staining when using different application methods with a brand regulated for clinical use in the study location.

Firstly, the results of the physical characterization study confirmed that the transmission profile of GreenGlo is lower compared to the other lissamine green brands tested, indicating a greater intensity of the dye in this fluid sample and a lower transmittance of light (i.e., darker dye solution) for all the spectrometer testing conditions (drop retention 0 s, 5 s or 10 s and ‘wash’ condition for 2 s using saline). These findings reinforce the results reported in a previous investigation where the concentration of GreenGlo was also found to be much higher than the remaining brands (GreenGlo 4.9% vs. OPGreen 3.4%, Biotech 0.9%, and Lissaver 0.5%) [4]. The high-resolution imaging suggests the fibre structure of the paper is similar, and while the strip thickness appears to vary, the difference between the two brands investigated in the in vivo study is unlikely to be a key factor. The drop weight from the LG strip was unaffected by the duration of the saline drop’s exposure to the strip tip for each of the brands. This implies there is no difference in fluid absorption into the strip or availability of lissamine green for application to the ocular surface between the strips, which could otherwise have affected the strip staining efficacy.

The concentration increased as the duration of retention of the saline on the strip tip increased from 0 to 5 or 10 s, aligning with the benefit of retaining the saline drop on the strip for at least 5 s before applying it to the ocular surface, as found in this and our previous study [7].

Secondly, the results of the in vivo study indicate that GreenGlo lissamine green strips provided better overall visibility of bulbar conjunctival staining with regard to both the quantity of visible punctate staining spots and staining intensity. The effect is most marked for the first 60 s until the excess drains. This finding is significant, particularly as Figure 4 and Figure 5 show that if a clinician was to use a single application 5 s after wetting a strip with a drop of saline, the lissamine green staining count (by, on average, two punctate spots out of the nine required to indicate a loss of homeostasis of the tear film for a dry eye diagnosis) and intensity would be much higher with GreenGlo compared to I-DEW. Previous work [7] suggests the optimal approach for bulbar conjunctival staining evaluation involves two applications (1 min apart) of a whole infused saline drop, each time resting on the same strip for 5 s in the same eye. Similarly, the present work also found staining intensity and punctate staining count were overall higher with a dual application of I-DEW as compared to a single application. However, the clinical significance of the differences, which are not significant at all time points post-instillation, are questionable with I-DEWS. This, however, reinforces the theory that applying the same strip twice should be the current recommended approach for using lissamine green strips for conjunctival staining evaluation and that this may be the case regardless of the brand/product used. However, it should be noted that a single drop of GreenGlo performed as well as the two applications of I-DEW, emphasizing that the application method and the dye brand are key considerations. It is important to note that two lissamine green strips (one for each eye, applied twice) would be used in a clinical setting. Interestingly, the use of two I-DEW lissamine green strips together, i.e., 3.0 mg of lissamine green in one application, did not provide the same level of staining, implying that the application method is more important for highlighting conjunctival staining than the weight of lissamine green in contact with the ocular surface.

The spot count and staining intensity values obtained is this study with the use of GreenGlo lissamine green strips are greater than the values obtained in the previous study investigating different application methods [7]. This implies that the severity of bulbar conjunctival staining in the participants of this study was likely greater, and this is reflected in the DEQ-5 scores from participants also being greater (this study 13.3 ± 5.5; previous study 11.3 ± 3.15) [7]. Overall, the results imply that clinicians and researchers who use lissamine green-impregnated strips should be aware that different brands can produce differing visibilities for similar conjunctival staining and thus may not be interchangeable. If a patient has had prior use of a specific brand or product recorded, the same should be used again with the product used listed on the patient record alongside the observed outcomes. In line with this, Delaveris et al. evaluated the variability in the performance of lissamine green strips when evaluating lid wiper staining and advised that clinicians must consider the variability in the performance of available lissamine green strips when interpreting negative findings in lid wiper staining, as they could relate to a more poorly-performing lissamine green strip rather than an actual staining absence [4]. Similarly, Lievens et al. also demonstrated that a single instillation of liquid lissamine green dye does not offer an adequate concentration of dye for lid wiper epitheliopathy identification [10].

The results also indicate that the greatest amount of lissamine green staining intensity is immediately after application. The number of apparent spots reduces rapidly until 60 s, when a more stable level is evidenced, suggesting that clinicians should image lissamine green staining at least 1 min after application. This finding is in line with a previous investigation focusing on optimizing the methodology and assessment of bulbar conjunctival lissamine green staining [7] and mapping the delay to view an optimal staining of the lid wiper region for epitheliopathy [11,12]. It should also be noted that the results indicate that the difference between the two products used in the in vivo study diminishes after the first minute. The amount of staining and staining intensity both reduce by a relative approximation of 50% compared to the peak amounts found immediately after application. It may be that the difference between products diminishes after this time, which may mean that clinicians may not observe a difference at this stage.

The results for the spot count and grading intensity appear to be relatively comparable, with the same application techniques producing greater staining visibility according to both measures. This was also seen in our prior study [7], providing reassurance that staining intensity may be appropriate for use in evaluating lissamine green staining.

This study had strengths in that it was multicentred, and data collection, grading evaluation, and statistical analysis were all performed independently to reduce sources of bias. However, a limitation of this study is that it did not investigate the combined use of fluorescein and lissamine green comprehensively, i.e., with more than a single method. As the use of both dyes is common in clinical practice, it would have been useful to determine how the visibility of staining using both agents could be improved. The results currently suggest that the use of fluorescein in combination with I-DEW lissamine green, even with two applications of lissamine green, provides a marginally inferior performance to using lissamine green alone for bulbar conjunctival staining. This highlights the need for future research into optimizing how best to use these dyes together. Additionally, the use of images captured on the slit lamp for evaluation is not that of gold standard or typical practice, where direct observation would be performed, and may give a better evaluation of staining present. However, image capture for evaluation is becoming more widely available to clinicians with automated objective grading software tools such as Advanced Ophthalmic Systems (AOS; Weybridge, UK). The use of images in this study allowed more than one person to grade the observed aspects to attain an average measure, as well as allowing for masked grading to minimize possible bias. Furthermore, this study did not include data on the experiences of participants themselves on the different techniques used in order to gather their comfort and clinical tolerance. Future studies which investigate the optimization of LG application methods should consider adding an evaluation on the tolerability of the different techniques as part of their investigations.

## 5. Conclusions

In conclusion, this study supports previous findings that two applications using the same lissamine green strip in the same eye, 1 min apart, after initially waiting 5 s after adding a single drop of saline to the strip, was the optimal method for increasing the visibility of conjunctival staining with I-DEW lissamine green. There appears to be a significant impact on the choice of product used, more so in the first 30–50 s, which resulted in variable staining visibility, indicating non-interchangeability. This difference may in part be due to the physical characteristics of the different products, as they appear to show different dye colouring and staining agent concentration.

## Data Availability

The data presented in this study may be available on request from the corresponding author due to consent restrictions.
